# The ethics of selling shares in your future income

**DOI:** 10.1177/1470594X251391208

**Published:** 2025-11-14

**Authors:** Friedemann Bieber, Huub Brouwer

**Affiliations:** 1 27217 University of Zurich, Zürich, Switzerland; 2 145168 Tilburg University, Tilburg, The Netherlands

**Keywords:** Debt-financing, equity-financing, moral limits to markets, equality of opportunity, relational egalitarianism, shares in people's future earnings, financial markets, right to credit

## Abstract

Could private markets in shares in people's future earnings be morally permissible, or even desirable? While markets for personal debt are widespread, markets for shares in the future earnings of individuals are not. In this article, we argue that from a normative point of view, these markets are worth taking seriously. We begin by surveying their potential upsides and set out a baseline model of such a market that is moderately regulated, but has a plausible claim to being economically viable. We then critically examine its moral permissibility by identifying what (i) moral limits to markets and what (ii) distributive and (iii) relational egalitarian concerns it could give rise to. We advance two claims. First, that in sufficiently idealised circumstances, markets in shares in people's future earnings are not only morally permissible but are desirable and in several ways superior to markets in personal debt. Second, that in non-ideal circumstances, these markets can give rise to serious distributive and relational concerns that make them morally objectionable, but this does not imply that they should always be prohibited. We conclude that markets in shares in people's future earnings have potential as a complement to existing markets and modes of public provisioning.

Could private markets in shares in people's future earnings be morally permissible, or even desirable? While markets for personal debt are widespread, markets for shares in the future earnings of individuals are not.^1^ Economists have explored the viability of such markets (Herbst and Hendren, 2024), but political philosophers have so far remained silent on the idea. This article offers an initial discussion of the ethics of selling shares in one's future income.

Our main question is as follows: can a market for shares in people's future income be morally permissible, and, if so, even desirable? To answer it, we proceed in six steps. We start by introducing the idea of a market for shares in people's future earnings and examine its promise. After that, we set out a proposal for regulating such a market: the *baseline model*. This baseline model will serve as the foundation for our normative examination. We then argue that if markets are regulated according to the baseline model, it is likely that they are economically viable. We subsequently critically examine the moral permissibility of these markets in a somewhat idealised setting over the course of three sections. We first apply the lens of the moral limits to markets literature, then consider distributive concerns, and finally examine relational egalitarian concerns. We argue that moral limits to markets and distributive concerns are defeasible, and that although relational egalitarian concerns are more pressing, they are still less serious than the ones raised by markets in personal debt. After that, we discuss to what extent our findings hold in less idealised settings. We argue that in such settings, markets in shares in people's future earnings can give rise to more serious distributive and relational concerns, which can make them morally objectionable. This does not always imply, however, that these markets should be forbidden. The final section concludes.

We want to be clear about what we will *not* be doing: we will *not* assess whether markets in shares in people's future earnings are the *best possible* solution for funding everyone's investments in education or entrepreneurial ventures, all things considered. It is possible to hold that such markets are permissible or even desirable and yet to also insist that the morally best way to stimulate investments in higher education and new businesses would be to implement tax and transfer policies, such as offering a capital grant to all young adults (cf. Robeyns, 2025; White, 2003: chp. 8), citizen stakeholding (cf. Ackerman and Alstott, 1999), a basic income (cf. Bidadanure, 2019; Van Parijs, 1995), or a combination of (some of) these (Piketty, 2020: chp. 17; White, 2004).^2^ Hence, we offer a *qualified defence* of markets in shares in people's future earnings. Such private markets have potential as complements to existing markets and modes of public provisioning.

## Promise and peril of a market in shares in one's future income

The idea of creating markets in shares in people's future earnings is not new. Milton Friedman suggested such a market to fund people's investment in higher education 70 years ago:

human capital investment necessarily involves much risk. The device adopted to meet the corresponding problem for other risky investments is equity investment … The counterpart for education would be to “buy” a share in an individual's earning prospects. (Friedman, 1955: 138, also cited in Herbst and Hendren, 2024: 2024)

The proposal we are evaluating in this paper is setting up markets in shares in people's future earnings in Friedman's spirit, but with a broader scope. Share issuers would be able to use these markets to fund an investment in higher education, entrepreneurial endeavours, housing, their health, and so on.

Markets in shares in people's future earnings can be set up in various ways, but the basic arrangement is this. Individuals who issue shares agree to share, every year, for a defined period of time, a certain percentage of their income with their shareholders: the dividend. They do so in exchange for an amount they receive at the moment of sale: the equity. All shares are issued through a corporate entity, and the share issuer agrees to channel all their income through this entity until the shares expire.^3^ How much dividend share issuers pay to their shareholders is determined by how many shares they issue and by their income level. If, for instance, someone sells a 10% share in their future income for the next 10 years for an equity of $100,000, then that person agrees to share 10% of their income each year with their shareholders. If that person were to earn $120,000 each year during the 10-year period, then investors would receive a dividend of $12,000 each year. If, instead, that person were to earn $1,200,000 each year, then investors would receive a dividend of $120,000 each year.

The promise of markets in shares in people's future earnings lies in the fact that, compared to debt markets, they shift the division of the risk of income shortfalls between those who obtain funding (the share issuer) and those who provide funding (the shareholder). In debt markets, those who obtain funding typically bear the risk of income shortfalls themselves: the amount they must repay stays the same, regardless of fluctuations in their income (unless they default on their debts). In equity-financing systems, people obtain funding and then pay out a dividend that varies depending on how much income they have. Exploring alternatives to debt-financing is important, because households in many countries are increasingly dependent on debt-financing,^4^ and there is growing evidence of negative consequences of household indebtedness.

At a societal level, markets in shares in people's future income hold promise for four different reasons. First, studies suggest that many societies seriously underinvest in human capital (World Bank, 2019: 49–67). In countries in which higher education is expensive, fear of indebtedness is part of the explanation for underinvestment in human capital (Herbst and Hendren, 2024). Allowing a market for shares in people's future earnings could help alleviate this concern, enabling younger persons to invest in their education and health without running the risk of defaulting on their debt if they have an earnings shortfall. Second, the rate of entrepreneurship is declining in countries such as the United States, which harms job and productivity growth (Decker et al., 2014): markets in shares in people's future earnings could stimulate entrepreneurial activity by allowing entrepreneurs to spread capital they collect across different entrepreneurial ventures.^5^ Third, such markets could help address intergenerational wealth inequalities, effectively bringing forward the point in time when access to equity is transferred between generations. Fourth, such markets could improve a sense of community, because by buying shares in others’ earnings, people become invested in each other's success. This is a notable difference to debt markets, in which creditors have little to gain from their debtors’ success (short of the avoidance of default).^6^

On a personal level, meanwhile, the markets hold promise in at least three ways (which partly overlap with their social benefits). First, such markets could liberate individuals from financial constraints, enhancing their financial freedom across their lifetime and improving their ability to design their own life plan.^7^ In debt markets, people are typically required to demonstrate that they have enough income before they are eligible for loans, especially in the case of mortgages. Markets in shares in people's future earnings, by contrast, only require the demonstration of a capacity to earn income in the future. Selling shares, then, is one way of pushing beyond current budget constraints, permitting the immediate pursuit of projects – be they educational, entrepreneurial, or otherwise – that would have been infeasible otherwise. Second, there is a growing body of evidence which suggests that indebtedness is bad for mental and physical health (Gathergood, 2012; Keese and Schmidtz, 2012). Markets in shares in people's future earnings could help people avoid these negative consequences of indebtedness. Third, markets in shares in people's future earnings could improve their capacity to realise the value of community in their personal lives, because their shareholders have an incentive to enable them to be successful.

But where there is promise, there is often also peril. Evidently, markets in shares in people's future earnings can give rise to serious normative concerns. Suppose, for instance, that shareholders were to get a say in central, income-affecting life decisions of share issuers – such as what degree to study or what business to set up. The underlying contract would then, if legally enforceable, not only significantly undermine the autonomy of the share issuer and invade their privacy but would also, arguably, place them in a state of (partial) slavery. This does not amount to a knock-down argument, however: the markets could be regulated in a way that ensures that shares are purely passive, and, consequently, shareholders have no say in any life decisions.

The interesting question is thus not whether markets in shares in people's future income are always permissible. They clearly are not. Instead, it is whether such markets could be set up in a way that renders them simultaneously economically beneficial and morally permissible, in both ideal and non-ideal circumstances. This is the question this article seeks to answer.

## The baseline model

To enable a moral evaluation of markets in shares in people's future earnings, we first set out a *baseline model*. This model captures a regulated version of the market that we believe could be economically viable and morally permissible. We divide the regulatory constraints into four categories: (i) income, (ii) eligibility, (iii) control, and (iv) investment. While we discuss these constraints in some detail, we do not claim that the market must be set up in *exactly* this way. Rather, we contend that regulation of the elements that we identify would be required and that our specifications constitute a reasonable starting point. The baseline model is summarised in Table 1.

**Table 1.** Baseline model.

I. Income and dividend	II. Eligibility	III. Control	IV. Investment
Dividend is based on income above a threshold (living wage) Income includes income from work, entrepreneurship, investments, and pensions Unrealised gains are excluded	Minimum age 18, no maximum age Initially shares in at most 20% of future income Additional shares can be issued in 10% increments every 5 years, to up to 50% No expiration date required (but possible)	Purely passive holdings, voting rights limited to vote on termination Information rights (income and value of assets and liabilities)	Share issuers cannot give away more than 10% of assets each year Share issuers can invest at most 5% of wealth above threshold in unregistered assets

### Income and dividend

The first set of regulations concerns a person's income and dividend payments. The baseline model stipulates an income threshold: any earnings below this threshold go to the share issuer and need not be shared through the dividend. This requirement ensures that selling shares in your future income does not place you in situations of dire economic necessity. We set the threshold to the local full-time employment living wage equivalent. Higher limits are certainly possible, but deepen moral hazard: individuals might avoid earning more than the limit to prevent having to share any of their income with shareholders.

Dividends are calculated on the basis of a person's income, where all labour income, investment income, and pension benefits are taken into account.^8^ Our baseline model does not, however, count unrealised gains and losses, that is, gains and losses that have accrued on paper due to a change in market prices, towards income.^9^ This adds to economic efficiency: it is likely that higher gains can be obtained when people are not forced to immediately realise unrealised gains, because it allows them to hold onto assets rather than forcing their sale.^10^

In the set-up we envision, share issuers would hand out dividends to external shareholders first and then pay taxes on the income that remains. The state should then separately tax external shareholders’ dividends, too.^11^

### Eligibility

The second set of regulations deals with eligibility. In the baseline model, individuals are allowed to sell shares in their future earnings from age 18 onwards. We chose this relatively young age because we believe that the benefits of markets in shares in people's future earnings arise, to a large extent, from share issuers using the equity they obtain to invest in education and entrepreneurial activities when they are young.

Initially, people are allowed to issue shares representing at most 20% of their future earnings. They can issue additional shares in 10% increments every five years, up to a maximum of 50%. This restriction gives them an incentive to use the equity to ends that improve their future earnings (and thereby also increase the equity they can obtain through future share issuance) and protects them from making decisions that they might come to regret, such as issuing many shares at low prices early on in life.

The baseline model allows people to issue shares *without* an expiration date, but does not require them to do so. We believe shares without an expiration date would be particularly attractive to investors and would help secure access for a large number of potential share issuers. If people do issue shares without an expiration date, they will in principle share their income with shareholders until they pass away. Once they do, any unrealised gains must be realised and will be divided up pro rata among shareholders, with the remaining capital stock going to the next of kin.

People can at any point decide to purchase back the shares they issued (at the current market price). They could also reach an agreement with shareholders to dissolve the arrangement, for instance when they become unfit for work for medical reasons.

### Control

The third set of regulations concerns control: what sorts of rights and powers are shareholders awarded? The baseline model unequivocally states that shares in people's future earnings are *purely passive* holdings. Shareholders do not obtain voting rights on any life decisions, dividend payouts, and so on. This averts the concern that a market in shares in people's future earnings would place share issuers in a state of (partial) slavery.

The sole voting rights shareholders get, concern the termination of the corporate entity through which people issue shares in their future earnings. This entity can only be terminated when a majority of *external* shareholders agrees to this – meaning that the shares of the share issuer don’t come with voting rights on this issue. The reason for this restriction is that otherwise the share issuer would have an incentive to terminate the corporate entity shortly after issuing shares.

To give shareholders some control in the absence of voting rights, and thus to help render the market viable, individuals selling shares must commit to annually reporting their personal income tax statement, an estimation of the value of their assets, and the tax statement of the corporate entity in which they sell shares. This means that shareholders can check whether individuals earn any income outside of the corporate entity from which they issue shares.

### Investment

The final set of regulations constrains how share issuers can deal with their assets. Share issuers cannot give away more than 10% of their assets to others each year. This rule aims to prevent shareholders from losing out on unrealised gains when assets are passed on to others, such as family or friends. Also, above a minimal threshold (we propose the equivalent of one year's income at the gross living wage level), share issuers can invest at most 5% of their wealth in unregistered assets such as art, clothes, and furniture. The reason for this restriction is that it is easier to hide such assets from the tax authority and hence to prevent investors from knowing about these assets too.^12^

Taken together, these regulations regarding (i) income and dividend, (ii) eligibility, (iii) control, and (iv) investment constitute the baseline model. It is important to reiterate that we do not claim that markets in the future income of individuals need to be set up exactly in line with the baseline model. As we will go on to argue, however, the baseline model does offer a promising starting point.

## Economic viability

It is evident, we believe, that a market adhering to the baseline model can in principle generate the social and personal benefits we have discussed in the first section. But is the baseline model economically viable? We believe that it is. Let's start to make this case by considering the United States, specifically the effects of investments in education there. But before we do so, we should be clear in stating that the baseline model allows share issuers to spend the equity they obtain on many different things, not just education (alternative uses could be investments in real estate or the founding of companies). The case of education is helpful here, however, because it is one about which statistical data is available.

In 2014 in the United States, the gross income of female college graduates during their working lifetime (between the ages of 20 and 69) was $2.73 million (in dollars valued at 2024 levels), compared to $0.68 million for women with lower than high school qualifications (Tamborini et al., 2015: Figure 2 and 1390). Assume, for simplicity, that lifetime earnings are spread out evenly over 40 years, from the age of 25 to 65, and that the protected living wage is $30,000. In this case, based on labour income alone, a 20% share in the average woman at age 20, who will go on to graduate from college, has a net present value of $124,000 if we assume a 4% real interest rate (or $86,000 if we assume a 6% real interest rate).^13^ These estimates provide lower bounds as they ignore returns on investment and pensions and the realisation of unrealised gains upon death.^14^

The sums involved show that despite the long investment horizon and a multi-year pre-revenue period when individuals are in college, these markets have the potential to be economically meaningful. The investment horizon for investments later in life, when individuals may seek capital to start a business or to invest in their home, would be shorter. This would reduce the present value of the shares.^15^ Yet this effect would be counterbalanced, at least to some extent, by the lower risk associated with these shares: a person's professional track record would give a clearer indication of their income potential. In addition, there would not be a prolonged wait period until the first dividend payment was made.

There is an important complication, of course: moral hazard. Because shareholders have no control over how their investment is used, a market would have to price in the risk that share issuers may simply take the money and run. Such a moral hazard may be thought to be quite severe: after all, only a few entrepreneurs have set up investment vehicles that resemble markets in shares in people's future earnings – and they have done so predominantly in sports and in tech. In sports, the investment corporation Fantex Holdings, Inc. started issuing shares in the brand of athletes in 2012, providing athletes with cash in exchange for a minority share in the future earnings derived from these brands (typically 10%) for a few years.^16^ In a similar vein, Big League Advance offers athletes up-front capital in exchange for a 1% to 15% share in their future earnings, which only has to be paid out if they get signed up in the major league of their sport.^17^ In (predominantly) tech, a group of investors launched a platform called Humanism.is, which enables equity investments in the future earnings of individuals and is currently open for applications.^18^ What stands out is that existing markets in shares in people's earnings are mostly in sectors with superstar dynamics in which ‘relatively small numbers of people earn enormous amounts of money and dominate the activities in which they engage’ (Rosen, 1981: 845).

We do not think, however, that the current rarity of markets in shares in people's future earnings and their concentration in sectors with superstar dynamics should be taken as evidence that widespread markets in shares in people's future income are economically unviable. This is because various legal changes would have to be made to enable equity markets to function in line with the baseline model and because the baseline model has not been tried yet. The first such change relates to the fact that it is not attractive at present to issue shares in your future earnings if you have an employment contract, because when you do, your income will be taxed twice: once when it is paid to you, and again when you pay part of it to shareholders through the corporate entity through which you have issued shares. As previously mentioned, in the set-up we envision, share issuers would hand out dividends to external shareholders first and then pay taxes on the income that remains. The legal change that needs to be made here is that the state would not tax the share issuer on the dividends they pay out, but would instead directly tax external shareholders on those dividends. Second, an important feature of our envisioned set-up is that share issuers do not get voting rights on the termination of the corporate entity through which they issue shares. Only external shareholders get voting rights on this issue, and they don’t get voting rights on anything else. Without such voting rights restrictions, there is a major risk that share issuers ‘take the money and run’ immediately after issuing shares – using their majority shares to outvote external shareholders on the termination of the corporate entity.^19^ Many countries have limits in place regarding the proportion of non-voting shares that companies can issue, which would prevent the implementation of the baseline model. Third, in the baseline model, individuals agree to channel *all* their income (not just income earned through one particular company, and not just labour income) through the corporate entity through which they issue shares. That restriction was not present in the real-world examples that we considered, but it does contribute to minimising moral hazard: if the restriction is not present, share issuers can avoid sharing income with their shareholders by earning non-labour income.

But even if markets in shares in people's future earnings are economically viable, they could face weighty moral objections. Investigating the morality of markets in shares in people's future earnings is the project of the remainder of this article. We will proceed in two steps. First, in the next three sections, we will assess the baseline model for an idealised society. Specifically, we assume that society does not feature deep inequalities (in wealth, income, or effective opportunity) or severe injustice (as in the denial of freedom to some, or severe forms of discrimination); that the market for selling shares in one's future earnings does not crowd out existing market or non-market options; and that the rules of the market are effectively enforced and robust across time. These are significant simplifications, but we believe that they are justified because they allow us to bring into focus the immediate concerns regarding introducing a market in shares in future income. After that, we discuss whether our findings also hold in non-idealised settings.

## Moral limits to markets concerns

Philosophers have put forward a range of accounts of the moral limits of markets, specifying the conditions under which markets become morally defective. In this section, we assess the baseline model in light of this literature. We focus on two central objections, the coercion and the corruption objection, and argue that neither applies to the baseline model in the idealised setting.^20^

### Coercion

The coercion objection holds that markets in certain goods are morally impermissible because even though people agree to sell the good in question, their agreement is, in fact, not voluntary (Sandel, 2012: 111). This could be because individuals are subject to (i) extreme vulnerability (e.g. due to destitution and a lack of alternative options) or because they suffer from (ii) weak agency (e.g. due to being in a limited epistemic position or lacking autonomy) (Satz, 2010: 94–99). Many markets contain some extreme vulnerability or weak agency. Consequently, defenders of accounts of moral limits to markets deem that a market is subject to the coercion objection only if these morally objectionable features arise to a significant degree, such as in the case of child labour or bonded labour (Satz, 2010: 98).

Markets in shares in people's future earnings that are regulated according to the baseline model are not vulnerable to the coercion objection. The income threshold creates an in-built safeguard against anyone issuing shares out of extreme vulnerability. This is because the shareholders can expect a dividend only if the share issuer ends up with an income above the living wage equivalent. So potential shareholders only have an incentive to purchase shares when they believe that a person has a corresponding earning potential. Although a person believed to have sufficient earning potential could be currently destitute, this is unlikely: precisely because they have such potential, they should have alternative options for generating an income.^21^

Moreover, there will be limited weak agency: the market rules are transparent, and the eligibility criteria ensure that people can only sell shares once they have reached a certain age, and then only gradually over time. This is not to deny that individuals could fail to fully understand the consequences of the decision to sell shares in their future income. But this applies equally to other decisions, like taking out loans, going into a particular profession, and having a child. Note, moreover, that decisions in this market have more limited consequences. First, they are reversible: share issuers can always purchase back their own shares. Second, downside risks are limited: share issuers remain entitled to keeping a living wage income to themselves, cannot sell shares in more than 20% of their income initially, and do not sign away any significant freedoms.^22^

We thus submit that the market for shares in people's future income is not vulnerable to the coercion objection, as it is ordinarily understood. Note that there is a second interpretation of the objection, however. This version holds that markets are objectionable if market transactions leave individuals susceptible to coercion *at a later stage* (even if the market transactions were not themselves coerced). Here, again, the income threshold ensures that even those who sell a large proportion of shares in their income will not end up in dire economic straits because of this. And the passive nature of shares guarantees that market transactions do not render a person subject to coercion. Share issuers sign away certain freedoms, such as the freedom to keep all the money they earn and the freedom to withhold information about their earnings from all others. But every contract involves some restriction on one's freedom, and these freedoms are not central: limiting them does not pose a threat to a person's agency or fundamental welfare interests.

### Corruption of meaning

The corruption objection^23^ holds that ‘certain moral and civic goods are diminished or corrupted if bought and sold’ (Sandel, 2012: 111; see also Anderson, 1990: 186 and 201). The value of an Olympic medal in gymnastics, for instance, would be greatly diminished if it were possible to purchase the medal rather than having to compete for it.

What counts as the corruption of moral and, especially, civic goods remains, to some extent, sensitive to cultural practices. But we hold that a market in shares in people's future earnings is unlikely to be vulnerable to the corruption objection. The primary reason for our view is that shares function as an impersonal investment vehicle: a payment made now is traded against the prospect of returns in the future. In this regard, the shares resemble other financial products, such as interest-bearing loans and futures in agriculture produce.^24^

In fact, the concern about corruption is less acute regarding shares in people's future earnings than for many widely accepted financial products. One worry is that markets may corrupt relationships among people by antagonising them. Historically, this has been a key objection to life insurance. Life insurance contracts, such as those for viaticals and life annuities, generate conflicting interests: some stand to gain economically from another person's timely death. By contrast, the market for shares in future income fosters an alignment of interests among contracting parties (as explained in the first two sections).

Even if one accepts this argument, one might still be concerned that these markets corrupt existing *personal* relationships. If family members or friends purchased shares in each other’s future income, this might still be thought to be corrupting, even if it aligned their interests better, because it results in a more instrumental and calculated way of relating to one another.

Although this concern could indeed arise, we do not believe it provides an objection to markets in shares in people's future earnings. First, the concern generalises. It occurs whenever people enter financial contracts with those to whom they are close – such as when siblings, romantic couples, or friends start a company together or borrow money from each other. In all these cases, a new, more transactional mode of interaction may undermine the more personal mode. If anything, this risk is comparatively restricted in markets in shares in people's future income, because of the clear and comprehensive terms of the arrangement and the fact that shares are purely passive holdings. In addition, the concern can only arise if people who are in close relationships decide to invest in each other's shares. They might choose not to. While it is permissible to borrow money from friends and family, for instance, some people deliberately forego this option.^25^ At best, then, this concern is about a specific set of agents engaging in this market and not at all unique to this market.

## Distributive concerns

A second set of concerns about markets in shares in people's future earnings relates to their distributive consequences. We focus on egalitarian theories of justice,^26^ because libertarian theories^27^ and desert and merit-based theories^28^ are unlikely to object to these markets. For the egalitarian, the main worry is that such markets undermine equality of opportunity.^29^ To establish whether they do, it is helpful to go through different definitions of equality of opportunity.

We focus on three interpretations, in increasing order of demandingness (Cohen, 2010). First, equality of opportunity can be understood in a purely formal sense: individuals enjoy equal opportunities to the extent that there are no formal barriers that exclude some on arbitrary grounds such as gender identity, caste, or race (formal equality of opportunity). Second, it can be understood in a more substantive sense as requiring that the state, in addition to realising formal equality of opportunity, corrects for unequal social circumstances, such as varying access to education and wealth (substantive equality of opportunity). Third, equality of opportunity can be understood in a socialist or luck egalitarian sense, as requiring that the state also corrects for the impact of any other unchosen characteristics and unchosen circumstances that can plausibly affect lifetime chances, such as a person's inborn talents (socialist equality of opportunity).

### Formal and substantive equality of opportunity

There is no reason to think that the baseline model would clash with formal equality of opportunity: the market is open to all, and arbitrary characteristics like gender identity and race are not legitimate grounds for formally excluding individuals from selling or buying shares.

Regarding substantive equality of opportunity, the situation is more complicated. In one regard, the market for shares in your future income is clearly beneficial: by introducing an additional way of obtaining financial resources, it renders opportunities that are less dependent on initial endowments, such as family wealth. This will be the case in particular for those with low initial endowments but high talent-based earning potential: the opportunity to sell shares in their future income extends their budget constraints. The market thereby reduces inequalities in option sets between them (group 2 in Table 2) and those who have high initial endowments and high or low talent-based earning potential (groups 1 and 3, respectively, in Table 2).

**Table 2.** Talent-based earning potential and initial endowment: Four groups.

(1) High earning potential, high initial endowment	(2) High earning potential, low initial endowment
(3) Low earning potential, high initial endowment	(4) Low earning potential, low initial endowment

Clearly, this positive effect extends only to those who are perceived to have a sufficiently high talent-based earning potential to effectively access the market. Investors have little incentive to buy shares in the future earnings of those who are expected to earn close to the living wage for their entire life, because the income threshold would prevent them from receiving any dividends. The market does not, therefore, improve opportunities for those who start out in the most disadvantaged (group 4 in Table 2). Markets in shares in people's future earnings thus eliminate the effect of unequal initial endowments for some, but not for all.

Moreover, there is the concern that such markets may, via two different routes, disproportionately benefit those with high initial endowments, thereby in fact reducing equality of opportunity among groups with equal talent-based earning potential (namely groups 1 and 2, and groups 3 and 4 in Table 2).

The first way through which the market could disproportionally benefit those with high initial endowments is that they may benefit from the new investment opportunity: they can now purchase shares in others who have a high earning potential and can benefit from their success. If the market increases the overall return on investments, this harms substantive equality of opportunity: initial endowment, which is a social circumstance, becomes more decisive. But we believe that any such effect will be quite limited. True, shares in people's future income must be attractive relative to other investment opportunities. As such, overall investment opportunities will likely improve. But competition will drive down expected returns and, given the large number of other investment opportunities, its effect on overall returns on investments will be limited.

The second way in which those with high initial endowments may disproportionally benefit is that they are likely to be better situated to sell shares in themselves. Put differently, endowments and earning potential may turn out to interact: in relation to a given level of talent, one can leverage one's endowments to sell one's shares at a higher price. This could be for a variety of reasons.^30^ Individuals from more advantaged backgrounds could (i) be more trained in presenting themselves in a way that is attractive to investors because of their upbringing, (ii) have an easier time finding buyers for their shares through family connections, and/or (iii) have an easier time finding employment through family connections.

This is a serious concern, but we believe it, too, will remain fairly limited. First, we have assumed that there are no deep inequalities in the distribution of income and wealth and no severe injustices in society, so differences in endowments will be limited and talent-based earning potential will likely be the most decisive determinant of overall earning potential. Second, there are good reasons not to invest in those with high endowments: because they anticipate large inheritances, which need not be shared with shareholders, they have less incentive to work hard. Third, there is a comparative point: individuals with higher initial endowments already have greater access to credit. Family connections and the ability to present oneself as a professional are highly beneficial for obtaining loans on favourable terms (as are highly paid jobs obtained through family connections). So, given the greater access to credit of those with large endowments, the market for shares in their future income would likely not benefit them much.^31^

Our result is mixed then. There is a clear route through which the market for shares in people's future income renders opportunities less sensitive to endowments and more sensitive to talent-based earning potential: it introduces a new possibility for obtaining equity. This possibility, however, is only available to those whose talents allow them to access the market as sellers of shares. At the same time, there are two other routes via which the market might render opportunities more sensitive to endowments: it introduces a new investment opportunity, and the equity that individuals can obtain by selling shares may not only vary according to their talent-based earning potential but also according to their initial endowment.

As we have argued, we believe that these negative effects will be fairly contained. But suppose that markets for shares in people's future income really does make opportunities more endowment-sensitive. The government could still intervene in a variety of ways to counterbalance this effect. It could act as a buyer, allowing individuals to obtain equity at a fixed minimum rate in exchange for paying a few percentage points extra tax on their income for the rest of their lives (or a shorter period) in lieu of dividend payments. It could also act as a subsidiser: by not taxing the dividends that individuals pay out to their shareholders (up to some threshold), selling shares would become an option for a wider range of people. These interventions could pre-empt or offset inegalitarian effects by adding a new publicly funded option for those with low initial endowments.^32^

### Socialist equality of opportunity

The third and most demanding notion of equality of opportunity we consider is socialist equality of opportunity. There are various accounts of socialist equality of opportunity (Segall, 2013). We focus here on a particularly influential one: luck egalitarianism. Luck egalitarianism holds, at the most general level, that justice requires that individuals should not be worse off ‘through no choice or fault of theirs’ (Parfit, 1995: 33). Nearly all variants of luck egalitarianism are committed to the claim that inequalities in the currency of justice (be it resources, wellbeing, or something else) are unjust when they are the result of brute luck rather than of option luck.

Option luck ‘is a matter of how deliberate and calculated gambles turn out – whether someone gains or losses through accepting an isolated risk he or she should have anticipated and might have declined’, whereas brute luck ‘is a matter of how risks fall out that are not in that sense deliberate gambles’ (Dworkin, 1981: 293).^33^ A state that furthers the luck egalitarian ideal, then, would be required to neutralise brute luck differences in the distribution of the currency of justice but not option luck differences.

Markets in shares in people's future income are attractive to luck egalitarians. First, they allow individuals with bad brute luck in the form of a low initial endowment to obtain equity by selling shares in themselves. These markets also offer a partial remedy to those who suffer from uncompensated brute bad luck later on in life by allowing a form of risk-sharing – by buying shares in others, individuals can voluntarily engage in a form of risk-pooling that was previously unavailable.

However, markets in shares in people's future earnings also raise three serious concerns from the luck egalitarian perspective. First, the price at which individuals can sell shares in themselves varies based on brute luck factors. In particular, those with a high talent-based earning potential will be able to sell their shares at a higher price. As a result, the market worsens the inequality in opportunity between groups 2 and 4 (in Table 2). Second, the opportunity for risk-sharing also varies based on brute luck factors. Only those who have initial capital (or can leverage their talents to secure equity) can use this form of risk-sharing. The mechanism thus improves equality of opportunity between groups 1 and 3 but worsens it between groups 3 and 4.

Overall, the market in shares in people's future earnings appears to leave the weakest further behind: those in Group 4 do not benefit from the opportunity to sell shares (due to their low earning potential) and suffer because the market produces greater returns on initial wealth (due to their low endowments).

We acknowledge that these are genuine and important concerns. However, our purpose in this paper is not to design policies that optimally advance equality of opportunity (or any other conception of social justice). We have the more modest aim of evaluating whether markets in shares in people's future earnings give rise to sufficiently serious distributive concerns that mean they are morally impermissible. We believe that this is not the case – not even on a luck egalitarian understanding of equality of opportunity. Although these markets fail to improve equality of opportunity for some (notably those with unequal talent-based earning potential) and could arguably worsen it for those with low initial endowments and low talent-based earning potential, this concern is not at all unique; it applies equally to debt-financing. Also, a possible solution would, again, be for the government to intervene and act as a buyer and/or subsidiser.

Interestingly, a further response is available to the defender of socialist equality of opportunity at this point. Some luck egalitarians stress the importance of the value of community. This value requires, according to Cohen (2010: 41), that people work with a particular motivation, namely ‘I produce in a spirit of commitment to my fellow human beings: I desire to serve them while being served by them’. Markets in shares in people's future earnings would promote this sense of community, because individuals literally become invested in each other's success – and consequently have an incentive to help each other.

## Relational concerns

We now turn our attention to a third set of concerns, namely that markets in shares in people may undermine our capacity for ‘relating to each other as equals’ (Lippert-Rasmussen, 2018: 61). Relational egalitarians differ regarding how they flesh out their ideal (Anderson, 1999; Lippert-Rasmussen, 2018; Schemmel, 2021). We focus on three demands that many relational egalitarian views have. First, a republican demand: relations among people should be free of oppression and domination (Anderson, 1999: 315; Lippert-Rasmussen, 2018: chp. 3.3–3.4.; Schemmel, 2021: chp. 3–4). Second, the no-social-hierarchies demand: this requires the distribution of recognition to be insensitive to native endowments and luck (Anderson, 1999: 312; Lippert-Rasmussen, 2018: chp. 3.6; Schemmel, 2021: chp. 6). Third, a more narrowly political demand: society should be structured in such a way that material inequalities do not undermine the functioning of democratic processes (Lippert-Rasmussen, 2018: chp. 9).^34^ The central question, for our purposes, is whether markets in shares in your future income would conflict with these demands.

Relational egalitarians could raise three concerns about the baseline model. First, that the arrangement imposes unilateral obligations and freedom restrictions on share issuers, such as the requirement to provide shareholders with extensive information and to limit the proportion of unregistered assets. These asymmetries may be thought to raise the spectre of asymmetrical privacy intrusions, oppression, and domination. Second, that the market creates a need to pitch oneself as a worthy investment opportunity, which may be a humiliating experience marked by a structural power asymmetry. This, too, might be thought to give rise to a risk of oppression and domination. Third, that the market effectively puts a price tag on individuals, with share prices indicating their perceived earning potential. This may be thought to exacerbate the perceived status inequality between (economic) winners and losers, and thus to undermine our ability to relate as democratic equals.^35^

While these are serious concerns for relational egalitarians, they are largely defeasible. The first point to note is that several features of the baseline model limit the severity of concerns about domination and oppression. The restriction to passive shareholdings severely reduces the extent to which asymmetrical obligations and freedoms can lead to domination or become demeaning. Moreover, the income threshold implies that individuals will not enter the market out of desperation. And finally, individuals can initially only issue shares in 20% of their future income, and can only ever issue shares in at most 50% of their future income, which limits the extent to which they can be dominated and oppressed by shareholders and the extent to which the market can amplify existing inequalities. The inequalities that the market creates, as well as the inequalities against which it operates, are thus such that they will not undermine people's ability to relate as equals, especially within a society without deep inequality in income and wealth and without severe injustices.

Regarding the effect of share prices on perceived status, we believe that this is a serious concern but that, again, it is restricted in several ways. First, one can in principle keep prices private by selling shares outside of a public exchange, for example, to family members or business partners.^36^ In this way, they need not become a dominating metric. Second, even where prices visibly diverge, this need not lead to a perception of status inequalities. The Nordic countries are most transparent in the world regarding incomes and tax returns yet they display a high degree of social cohesion and a strong sense of democratic equality. In fact, transparency could have the opposite effect: if share prices unmask significant inequalities in prospective earnings (e.g. between men and women), this might call into question how deserved these earnings are, prompt a clearer disentanglement of perceived status and income, or even trigger distributive political reforms that reduce the disparity in earnings.

Admittedly, how the market would affect perceptions of status is ultimately an empirical question. At least for the society under consideration here, which is assumed to be comparatively equal, we do not think that it would plausibly undermine the conditions of democratic equality.

But suppose one were to remain sceptical of this claim. Even then, we believe, one would have to accept that analogous concerns apply to other markets, like those for debt, rental housing, and labour. To wit: those who owe debt incur a range of obligations, principally of servicing their debt; those who rent an apartment have less freedom to remodel it and may be faced with sudden and substantial rent increases or eviction; and those seeking a job must sell themselves as attractive candidates. In each case, moreover, the divide between those controlling and those seeking resources creates a potential status inequality. Especially for debt markets, these concerns are, if anything, more pressing. This is because unlike shareholders in the baseline model, creditors typically enjoy far-reaching rights to repayment, and otherwise to seising the debtor's assets.^37^ Moreover, creditors are often free to raise interest rates,^38^ which can create a spike in repayment costs and make it unclear how much the debt will cost overall. By selling shares in your future income, in contrast to taking out debt, you do not put your belongings at risk, share the risk of revenue shortfalls with your shareholders, and have a more predictable schedule of repayment (simply a percentage of your income). These elements not only reduce concerns about destitution, domination, and weak agency. They also generate more equal relationships: equity-financing creates partners who share the risk of income shortfalls.^39^

Of course, the fact that other markets fare even worse in a certain regard does not imply that the market for shares in people's future earnings is morally acceptable. But the comparative assessment implies that if the relational egalitarian wants to object to the market for shares in your future income on the grounds noted above, then they are also committed to objecting to other, broadly accepted markets. And it is important to stress that these markets interact: access to equity gives you the option of refusing work that undermines your autonomy, is badly paid or demeaning, and/or invades your privacy; it also provides you with funds so that you can move from renting to owning a home; and, perhaps most importantly, it reduces your reliance on debt-financing. This means that even if the relational egalitarian has residual concerns about the baseline model of a market for shares in your future income, they may find that even by their own standards, having it is preferable, all things considered, to not having it.

While it is impossible to predict with certainty the effect of the market for shares in people's future earnings on relationships among people generally, we believe that the relational egalitarian lacks grounds for rejecting the baseline model outright. In fact, as we have indicated, there are good reasons to think that relational egalitarians should favour such markets because they reduce reliance on other, plausibly inferior alternative markets.

## Markets for shares in people's future income in non-ideal circumstances

Our analysis of the baseline model of markets in shares in your future income has relied on three simplifying assumptions: (1) that society does not feature deep inequalities (in wealth, income, and effective opportunity) nor severe injustices (like the denial of freedom to some, or severe forms of discrimination); (2) that the market for selling shares in one's future earnings does not crowd out existing market or non-market options; and (3) that the rules of the market are effectively enforced and robust across time.

In this section, we discuss the implications of relaxing the first assumption: that society does not feature deep inequalities. We focus on this assumption here for space reasons. It is, we believe, the most central concern given the paper's focus on the value of equality.^40^ Our finding is twofold. First, in the absence of this idealising assumption, the market *can* become *prima facie* morally impermissible. Second, this does not imply that these markets should be forbidden*.*

To assess whether relaxing the assumption that there is an absence of deep inequality and severe injustice in society could make the concerns we have discussed more pressing, it is helpful to consider a case in which there is not only deep inequality but also this inequality is the result of an injustice. Suppose that the lifetime earnings of women are substantially less than those of men and that this difference is *exclusively* the result of unjust wage discrimination.

To make this vivid, return to the estimated lifetime earnings we noted when assessing the economic viability of markets in people's future earnings. Our figures were for women. But there is a substantial gender discrepancy. In 2014, male college graduates had an estimated working lifetime (between the ages of 20 and 69) gross income of $4.47 million (in dollars valued at 2024 levels), compared to $2.73 million for female college graduates (Tamborini et al. 2015: Figure 2 and 1390). If lifetime earnings are spread out evenly over the 40 years from age 25 to 65 and the protected living wage is $30,000, then a 20% share in the earnings of the average man at age 20 who will go on to graduate from college has more than twice the net present value of a 20% share in the analogous woman: $260,000 compared to $124,000, assuming a 4% real interest rate.^41^

Assume, for the purposes of argument, that the difference in lifetime earnings between men and women is exclusively the result of unfair gender wage discrimination. Would the injustice of unequal lifetime incomes render a market in shares in people's future earnings impermissible?

As far as arguments about the moral limits to markets are concerned, the market would still not be subject to the coercion and corruption objections. This is because the safeguards built into the baseline model would continue to prevent anyone from issuing shares because they were destitute (the living wage threshold would prevent this), and the existence of gender discrimination would not make it more likely that the meaning of important moral and civic goods would be undermined by individuals selling shares in their future earnings.

The market would, however, now give rise to more serious distributive concerns. Although formal equality of opportunity would still be respected because women are not formally excluded from the possibility of buying and selling shares, the market would undermine substantive and socialist equality of opportunity. The difference in the price at which men and women could sell shares would no longer reflect (only) natural talents and personal choices, but also gender wage discrimination. Consequently, the market would give men greater access to capital.^42^ Not only would men be able to sell shares at a higher price, but a greater percentage of men would be able to do so. As a result of this unequal access to capital, men would have more educational and entrepreneurial opportunities, which could amplify inequality in lifetime earnings between men and women.

From the perspective of relational equality, the presence of discrimination would be even more concerning. A market for shares in people's future earnings would threaten to intensify objectionable status hierarchies between men and women. Women would be able to raise less equity than men by selling shares, which could plausibly create an image of them being less valuable in the economy, and to society more generally. And women's structurally more limited access to capital would create various opportunities for men to dominate women. Women would, for instance, find it more difficult to resign from a job or to leave harmful personal relationships because they would have less capital to fall back on.

These concerns are very serious and are sufficient to deem markets in shares in people's future earnings *prima facie* morally impermissible. But we believe that it does not necessarily follow that these markets should also be forbidden.^43^ It may, *all things considered*, still be preferable to allow them.

The key reason for this is that markets in shares in a person's future income provide access to a crucial good: a form of credit. In recent years, several philosophers have argued that individuals in capitalist societies have a right to credit (Brownlee and Stemplowska, 2015; Hudon, 2009; Meyer, 2018). This right entails that individuals have a right to borrow money at reasonable rates. Such a right could be grounded in various ways. It can be argued that it follows from a more general human right to financial inclusion (Brownlee and Stemplowska, 2015), or that it is justified by the exclusionary nature of private property, which restricts control rights over the means of production to owners (Meyer, 2018). If there is a right to credit, then banning a market in shares in people's future earnings in capitalist societies could lead to rights violations, especially if the remaining alternatives for obtaining credit are morally worse.

We believe that markets in shares in people's future earnings remain superior to the main alternative of debt-financing.^44^ In one sense, the two are equal: women in the imagined society who suffer because of wage discrimination would be able to borrow less and at higher costs, so debt markets would be vulnerable to the same charge that they undermine substantive and socialist equality of opportunity as well as relational equality. But unlike debtors, shareholders have a strong financial incentive to work together with share issuers to ensure that their income will rise. When there is significant labour market discrimination, this can give shareholders an incentive to help fight such discrimination to increase their returns. The markets we propose could create incentives to ameliorate the situation. Perhaps, however, we are too optimistic here: to really establish how debt and equity-financing would perform in societies with deep inequalities requires empirical work, and it could turn out that debt markets would in fact be superior.^45^

Our claim here is limited. We have not attempted to assess what would be the best way to fund people's investment in higher education and entrepreneurial endeavours, which may well involve direct provision or public subsidies. Nor have we considered all possible kinds of injustices: we do not rule out the possibility that the introduction of the baseline model could in some situations render a bad situation even worse. Generalising to other injustices is difficult. Considering whether these markets are permissible in situations with high inequality and/or severe injustices requires careful examination of the situation at hand. Our tentative conclusion, then, is that in situations of deep inequality and injustices, distributive and relational concerns can render these markets morally objectionable, but this need not imply that they should be prohibited.

## Conclusion

History is rife with markets in financial products that were once ostracised but have long become widely accepted, such as the market for interest-bearing loans (Graeber, 2014; Le Goff, 1988), the market for life insurance (Rotman Zelizer, 2017; see also Bouk, 2018), and the market for futures in agricultural commodities (Levy, 2012). In this article, we have offered a moral assessment of a seemingly outlandish market that has not been discussed by philosophers: a market for shares in people's future income. We have argued that although such a market may initially appear radical and morally objectionable, there are good reasons to take it seriously when it is regulated properly.

Proper regulation amounts to a balancing act, ensuring that markets are simultaneously economically viable and morally desirable. We have specified a baseline regulatory model for markets in shares in people's future earnings that, we believe, successfully strikes this balance. We have argued that, in the context of a moderately equal society, a market regulated according to the baseline model is not only morally permissible but is also desirable and superior in several ways to markets in personal debt. In non-ideal circumstances, such a market can give rise to grave distributional and relational concerns and *can* become *prima facie* morally impermissible. Even so, we have maintained, it need not follow that these markets should be forbidden.

We hope to have shown here that well-regulated markets in shares in people's future earnings are worth taking seriously. They need not raise moral concerns that are more significant than those prompted by widely accepted markets, such as markets for debt, labour, and rental housing. In fact, this form of equity-financing has some important advantages compared to these widely accepted markets, because it amounts to a risk-sharing arrangement. This is not, by any means, to say that markets in shares in people's future income are a panacea or should be preferred to public provision or redistribution. But it does show, we contend, that they warrant consideration as a complement to existing markets and modes of public provisioning.

## Notes

1. Several countries have government-backed and/or government-funded student loans, business loans, and income insurance schemes in place. Unless otherwise mentioned, we are concerned with *private* markets in student loans, business loans, and income protection schemes.

2. Another possibility would be the implementation of government-subsidised markets in people's future earnings. We return to this possibility in the section on distributive concerns.

3. Allowing individuals to issue shares through a corporate entity is likely to be the most straightforward set-up. One reason for this is that it can avoid double income taxation. Another is that this creates a separation between the personal assets of an individual and those of the corporate entity through which they issue shares, making clear that shares do not entail ownership rights for individuals. The corporate entity can be set up in many ways, but its precise set-up is not the focus of this article. One example of how it could be set up within the current regulation of the United States is offered by Humanism.is; see https://public.humanism.is/faq (in particular the section titled ‘Legal’).

4. See the IMF's data mapper for household debt-to-GDP ratios: https://www.imf.org/external/datamapper/HH_LS@GDD/CAN/GBR/USA/DEU/FRA/JPN/SWE?year=2023.

5. If the creation of this additional investment market increases returns across asset types (by redirecting funds to investments with higher returns), it could also incentivise higher savings.

6. A striking example here is that mortgage lenders impose a penalty on those who wish to repay their premium too swiftly.

7. On freedom across lifetimes, see Schmidt (2017). On intrapersonal justice and the ability to design your own life plan, see Daniels (1985: 97–99).

8. We follow the United States Internal Revenue Service (IRS) in defining labour income as consisting of wages, salaries, and tips – but not pensions, welfare benefits, or social security benefits – and investment income as ‘interest, dividends, capital gains, rental and royalty income, non-qualified annuities, income from businesses involved in trading of financial instruments or commodities and businesses that are passive activities to the taxpayer’ (IRS, 2024).

9. To further improve economic viability, it would be possible to add a fourth constraint here, according to which share issuers need to sell assets with unrealised gains if their earnings are below the income threshold for payouts for a period of five years. We do not believe such a restriction to be essential, however, because share issuers already have various incentives to increase their income.

10. We think gifts and inheritances, as well as subsequent gains from investing them in separate accounts, should also be excluded. Otherwise, people's share prices would no longer reflect only their earning potential but also the wealth of their family and friends. This would undermine economic efficiency as the market would no longer channel capital to those who are likely to invest it in ways that generate higher expected earnings.

11. In addition, the state could choose to tax the initial revenue raised through the sale of shares, be it through a stamp duty (i.e. a fee for issuing stock) or by taxing the receipts as income.

12. Why impose such restrictions while granting people the freedom to choose their occupation and how much to work (if at all)? Our underlying rationale here is that freedom of occupational choice is more important than freedom regarding how to spend the money you have earned. Restricting people's occupational choice is more likely to violate equality of opportunity and relational equality than restricting how much money they can hold in unregistered assets and how much they can give away every year.

13. Our calculation ignores three complications. First, that incomes tend to rise with age, so returns will skew towards later years, which reduces the present value of shares. Second, that many individuals will earn below the living wage in some years; this implies that a larger proportion of income will be split with shareholders, thus increasing the present value of shares. Third, that the living wage might need to be adjusted to account for family size and, possibly, for geographical location.

14. The estimated lifetime gross incomes for male college graduates are higher. We will discuss this difference and its implications for our argument in the markets for shares in people's future income in the section on non-ideal concerns.

15. At least in so far as income from work or pension claims is concerned (the same does not hold for investment income), shares decay: the older a person is, the lower their expected remaining income is.

16. For details, see https://www.sec.gov/Archives/edgar/data/1573683/000104746913009713/a2216998zs-1.htm.

17. Baseball player Padres Tatis has is suing Big League Advance at the time of writing to challenge a deal he signed with them at the age of 17 (see Associated Press, 2025).

18. For a profile of two of the tech entrepreneurs behind this platform, see Heller (2022).

19. For an overview of the literature on different voting right regimes and their effects on company performance, see Adams and Ferreira (2008).

20. A third concern sometimes raised in the moral limits to markets literature is that markets undermine people's capacity to relate as equals (Satz, 2010: chp. 4). We take this concern very seriously and have devoted a separate section to it: the section on relational concerns.

21. Admittedly, we can imagine situations in which a person, though not destitute herself, needs money to care for friends or relatives, and is in this sense vulnerable and pushed to sell shares. But in this case, the problem is rooted in the wider social circumstances and extends to other markets. For example, this person might likewise be pressured to take out a loan, sell her belongings, or take on a dangerous job.

22. In these regards, the case differs starkly from, for example, the decision to engage in surrogate pregnancy work. The decision to become a surrogate is not easily reversible and carries a risk of devastating physical and psychological consequences (Satz, 2010: chp. 5).

23. To be precise, the concern with corruption is arguably more about commodification than marketisation; on this distinction, see Agmon (2025).

24. Admittedly, markets for these products were once deemed corrupting, often on religious grounds. Interest-bearing loans were considered to corrupt the proper function of money (as a medium of exchange) and time itself (in making an ownership claim on time) (Graeber, 2014). Futures in agricultural commodities were said to corrupt the sacred nature of food, turning it into an object of speculation (Levy, 2012: chp. 7). But today these markets are widely accepted: the concerns are mostly seen as misguided while their economic value is broadly acknowledged.

25. In fact, the creation of an impersonal market for shares in future income might reduce reliance on more informal arrangements, such as relatives putting together a loan to cover the costs of attending university. On the choice of whether to borrow money within families, see Gustke (2015).

26. We want to acknowledge our positionality here: our focus is on central theories of justice in Western analytic political philosophy, our area of specialisation. There are, of course, many other valuable theories in other traditions (such as communalism) that could give rise to different concerns about these markets. These are important topics for future research.

27. For libertarians, state restrictions on the sale of such shares amount to an interference with a person's right to self-ownership, in particular their control rights over the use of their body and the right to transfer these control rights to others. Their view is that the state is not justified in interfering with this market. We believe that this result extends to left libertarian theories – at least versions that hold that individuals have full ownership rights in their natural talents (for discussion on left libertarianism and such ownership rights, see Quong, 2011).

28. As Daniels points out, meritocrats typically hold that we should avoid talent being wasted because of people lacking sufficient opportunities to develop it (1978: 217). A market for shares in people's future earnings can clearly improve people's ability to make the most of their talents: share issuers can attain additional equity, which allows them to invest in education and training.

29. While one might also be concerned that the markets increase outcome inequality, we believe that this is a concern primarily where outcome inequality is a result of unequal opportunities, especially because we assume a compressed distribution of income and wealth to begin with.

30. We have excluded one obvious concern through the market's set-up: inheritances and gifts do not count as income and will thus not be shared with shareholders (see footnote 10).

31. We discuss the comparison with debt markets in greater detail in the section on non-ideal concerns.

32. Government-funded interventions in these markets are, of course, also possible when substantive equality of opportunity has not, on balance, been affected or has improved. We don’t get into this here, because our focus is on the moral permissibility of private markets in people's future income.

33. Although Dworkin originally introduced this distinction, he denied that he was, in fact, a luck egalitarian (2003: 190; see Arneson, 2018 for discussion).

34. On this point, Anderson (1999) offers a more minimal account, and primarily insists on the importance of sufficiency. Schemmel (2021) argues that relational egalitarianism requires stringent limits to material inequality.

35. On meritocratic hubris, and the tyranny of meritocratic thinking more generally, see Sandel (2020).

36. Note, however, that to be legally binding, such private sales would still have to abide by the rules of the baseline model.

37. They bear the risk that the debtor may default, but not the risk of revenue shortfalls.

38. This is often true for student debt; and mortgages often have fixed rates for only part of their duration.

39. In fact, those criticising debt markets frequently propose introducing forms of risk-sharing that move them closer to equity markets (see, e.g. Herzog, 2017).

40. Future research would be required to evaluate the effects of relaxing the other two concerns. With regard to the crowding-out assumption, it is important to note that even if some crowding-out does occur, it need not constitute a harm; and even if it does, it may be possible to rectify this by other means than prohibiting markets in people's future incomes. With respect to the effective enforcement assumption, it is important to evaluate not whether markets in shares in people's future earnings become problematic when enforcement is ineffective (they will), but whether enforcement in such markets will be especially difficult and/or likely to be ineffective compared to in markets that fulfil the need for credit.

41. The actual difference may even be higher than this. Because of their higher labour incomes, men will have more opportunity to save and invest than women.

42. Within each gender group, the market would still, however, increase substantive equality of opportunity, namely by reducing the relevance of initial capital endowments.

43. Satz (2010: chp. 4) provides a helpful discussion of possible policy responses in situations in which markets are noxious, suggesting various alternatives to banning markets that could help reduce noxiousness. She stresses that when considering whether to ban a market, it is important to consider not only the harms of the market itself but also the harms that may arise if the market is forbidden.

44. Evidently, it would be better still if the government could end gender discrimination in the labour market.

45. One factor that we have not discussed so far is attitudes to risk. If women are, on average, less likely to default on loans than men because they are more prudent, then it may in fact be easier for them to obtain a loan than to obtain equity by issuing shares.
